# GHRL as a prognostic biomarker correlated with immune infiltrates and progression of precancerous lesions in gastric cancer

**DOI:** 10.3389/fonc.2023.1142017

**Published:** 2023-07-04

**Authors:** Jinyun Wang, Dingwei Liu, Yong Xie

**Affiliations:** Department of Gastroenterology, Digestive Disease Hospital, The First Affiliated Hospital of Nanchang University, Nanchang, Jiangxi, China

**Keywords:** GHRL, gastric cancer, prognosis, tumor immune microenvironment, Correa’ cascade

## Abstract

**Objective:**

Ghrelin is a protein that regulate appetite and energy balance in the human body, which is encoded by the ghrelin prepropeptide gene (GHRL). GHRL is linked with carcinogenesis and immune regulation. However, the correlation of GHRL to prognosis and tumor-infiltrating lymphocytes in gastric cancer (GC) remains unclear.

**Methods:**

In this study, we assessed the transcriptional expression, prognosis, and different clinicopathological features about GHRL and the correlation between GHRL and tumor infiltration immune cells in GC patients based on the data published in the following databases: TIMER, GEPIA, GEO, STRING, UALCAN, TISIDB, and Kaplan–Meier Plotter. Furthermore, R software analysis for GC Correa’ cascade was also provided. Finally, GHRL expression in GC tissues was assayed using quantitative real-time polymerase chain reaction and immunohistochemistry.

**Results:**

We found that GHRL expression in GC samples was lower than in normal samples and verified by quantitative PCR (qPCR) and immunohistochemistry. However, sample type, cancer stage, and worse survival were correlated to high GHRL expression. We also found that the expression of GHRL in dysplasia was significantly lower than that in CNAG and in GC. High GHRL expression was connected with immunomodulators, chemokines, and infiltrating levels of B cells, CD8+ T cells, CD4+ T cells, macrophages, neutrophils, and dendritic cells in GC.

**Conclusions:**

GHRL is a prognostic biomarker for GC patients, and it is correlated with progression of precancerous lesions in GC. It might lead to poor prognosis by regulating tumor immune microenvironment. Studies are important to explore therapeutic targeting GHRL in the future.

## Introduction

Gastric cancer (GC) is the fifth most common cancer and the third most common cause of cancer death globally (1). GC patients are usually at late stages when diagnosed, thus losing the opportunity for surgery and with the poor prognosis (2). Currently, immunotherapy has become an effective treatment in GC (1, 3–7). However, not all GC patients benefit from immunotherapy because of the different immune microenvironment of tumor (8, 9); it is urgent to seek immune-related biomarkers in GC.

Ghrelin was discovered and named by Kojima in 1999 (10), which was a 28-amino acid polypeptide secreted by the stomach and released into the blood, acting on the growth hormone secretagogue receptor (GHSR), thereby promoting the release of growth hormone. Ghrelin exists in the human body in two forms, active (acylated-ghrelin) and inactive (deacylated ghrelin). Ghrelin was encoded by the ghrelin prepropeptide gene (GHRL) (11). Dixit et al. found that ghrelin and its receptor (GHSR) were expressed in T cells and monocytes; ghrelin inhibited the expression of proinflammatory cytokines tumor necrosis factor alpha (TNF-a) and interleukin (IL)-6 and inhibited inflammatory reactions (12). It has been confirmed that the stomach was indeed not the only source of ghrelin (13); hypothalamus, hippocampus, pituitary gland, cortex, small intestine, and pancreas can also secrete a small amount of ghrelin (14–16), GHRL plays important roles in the development of tumors, and aberrant GHRL expression has been found in various cancers, such as breast cancer (17), lung cancer (18), bladder cancer (19), and adrenal cancer (20). At present, there were some researchers who found that ghrelin affects cancer cell proliferation, migration, and invasion through different signaling pathways (21–24). However, the possible mechanisms of GHRL about tumor development, the GHRL expression in different stages of GC Correa’ cascade, and immune engagement in GC has not been well understood before.

In this study, the GHRL expression and its connection to prognosis in GC were presented via diverse databases including the Gene Expression Profiling Interaction Analysis2.0 (GEPIA2), UALCAN, STRING, Kaplan–Meier (KM) plotter, and TISIDB datasets. Furthermore, we used R software to further analyze the dynamic changes in GHRL in different pathological stages of GC Correa’ cascade (25); the Tumor Immune Estimation Resource2.0 (TIMER2.0) was used to investigate the relationship of GHRL with immune-related cells in the tumor microenvironments. In addition, GHRL expression in GC tissues was assayed using quantitative real-time polymerase chain reaction and immunohistochemistry. This study uncovered the critical involvement of GHRL in GC, Correa’ cascade, and the possible mechanisms via which GHRL may regulate tumor-infiltrating immune cells.

## Methods

We followed the methods of Junchang Zhang et al., 2022 (26). Furthermore, GEO analysis were also involved in this study for comprehensive bioinformatics analysis.

### Tumor Immune Estimation Resource database analysis

The Tumor Immune Estimation Resource (TIMER2.0) is a platform to analyze immune infiltration in various cancers (https://timer.cistrome.org/)^1^ (27). We investigated GHRL expression in GC and the relationship between the expression of GHRL and TILs. Furthermore, the relationship between GHRL expression with gene markers of TILs, including markers of CD8+/CD4+ T cells, B cells, monocytes, TAMs, M1 macrophages, M2 macrophages, natural killer (NK) cells, neutrophils, and dendritic cells (DCs) has been analyzed.

### Gene Expression Profiling Interaction Analysis

The Gene Expression Profiling Interactive Analysis2.0 (GEPIA2) database (http://gepia.cancer-pku.cn/index.html) ^2^is a platform that obtains the data from TCGA and The Genotype–Tissue Expression (GTEx) databases (28). In this study, we used the GEPIA data source to analyze the expression and survival of GHRL in GC.

### UALCAN analysis

In this study, we used the UALCAN website (29) (https://ualcan.path.uab.edu/)^3^ to examine the GHRL expression level from major clinical features such as tissue type (healthy/tumor), *Helicobacter pylori* status, and GC stage (stages 1–4).

### GEO analysis

To further validate the expression of GHRL in GC Correa’ cascade, GEO microarray series (GSE55696, GSE130823, GSE87666, GSE116312, GSE106656, GSE160116, GSE5081, and GSE78523) containing STAD tumor and non-tumor samples were obtained from the National Center for Biotechnology Information’s (NCBI) Gene Expression Omnibus (GEO, https://www.ncbi.nlm.nih.gov/geo/)^4^.

### Kaplan–Meier plotter

The prognostic significance of GHRL in GC, including overall survival (OS) and post-progression survival (PPS), was investigated using the KM plotter (30) (http://kmplot.com/analysis/)^5^.

### TISIDB

TISIDB (http://cis.hku.hk/TISIDB/index.php)^6^ is an online platform that combines various heterogeneous data to find tumor and immune system interactions (31). In this research, TISIDB was used to investigate the association of GHRL with TILs, immunostimulators, immunoinhibitors, chemokines, and receptors in GC.

### Protein–protein interaction network construction and enrichment analysis

STRING (http://string-db.org)^7^ is an online database for the prediction of protein interaction relationships (32). The GHRL-related genes were displayed in the STRING website. Then, we performed enrichment analysis using Metascape (https://metascape.org/)^8^.

### Tissue samples

In this study, paired GC tissues and adjacent non-cancer tissues were acquired from the First Affiliated Hospital of Nanchang University. Nine pairs of fresh GC tissues and adjacent non-cancer tissues were cryopreserved and were used for the quantitative analysis of the expression of GHRL by quantitative real-time PCR (qRT-PCR) and immunohistochemistry. All patients signed an informed consent form. The Ethics Committee approved the study of the First Affiliated Hospital of Nanchang University (Ethical Application Ref: 2021006).

### qRT-PCR

The total RNA of GC tissues and adjacent non-cancer tissues was extracted using RNA extraction kit (Yeasen Biotechnology, China). cDNA synthesis was performed using the Hifair III 1st Strand cDNA Synthesis SuperMix (Yeasen Biotechnology, China). qRT-PCR was performed using the SYBR Primix Ex Taq™ II (Tiangen Biotechnology, Germany) on ABI-7500 instrument (Applied Biosystems, United States). Actin was used as an internal reference gene, and the 2^−△△Ct^ method was used to compare the expression of GHRL. Primers used for qRT-PCR were as follows: ACTIN, 5′-CTCCACCCTGGCCTCGCTGT-3′ (F), 5′-GCTGTCACCTTCGTTCC-3′(R); GHRL, 5′-TACTACTCTCCACGCCC-3′ (F), 5′-AGGGGCCATCCACAGTCTTC-3′ (R).

### Immunohistochemistry

Immunohistochemistry (IHC) was performed to investigate the expression of GHRL. IHC staining of these specimens were conducted as previously described (33). Anti-GHRL (Santa Cruz Biotechnology, Dallas, TX, USA, sc-293422) were used for IHC staining.

### Statistical analysis

HR and *p*-values were described using log-rank test. Spearman’s correlation coefficient was used to analyze the connection of GHRL expression with immune infiltration levels, immunostimulators, immunoinhibitors, chemokines, and receptors. Statistical significance was defined as *p* < 0.05.

## Results

### GHRL expression in GC

**Figure 1:**
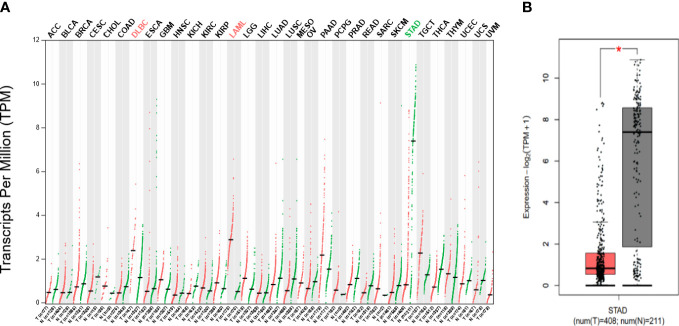
**(A)** Difference in GHRL expression in pan-carcinoma (GEPIA), low expression in STAD at RNA level **(B)**. Red mark represents that GHRL expression in tumor is higher than adjacent issue; green mark represents that GHRL expression in tumor is lower than that in adjacent issue. *p < 0.05.

According to the GEPIA database, we discovered that GHRL expression level was increased in diffuse large b-cell lymphoma and acute myeloid leukemia but was decreased in stomach adenocarcinoma (STAD) compared with healthy tissues. **Figures 1A, B** show the findings.

### Relationship between GHRL expression and GC patient clinical pathology

**Figure 2:**
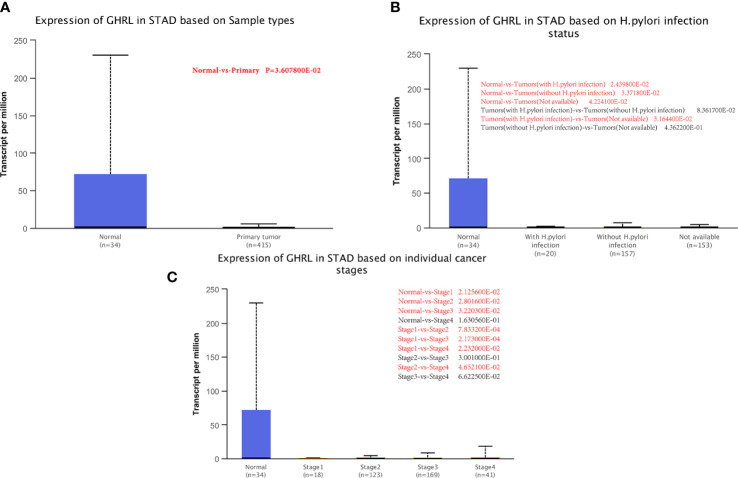
Correlation between GHRL mRNA expression level and clinicopathological parameters of GC through the UALCAN database. **(A)** Sample type (normal/primary tumor). **(B)** (*H*) *pylori* status. **(C)** Cancer stage (stage 1–4). N, normal; P, primary tumor; S1, stage 1; S2, stage 2; S3, stage 3; S4, stage 4; STAD, stomach adenocarcinoma. Red mark represents a statistically significant difference between groups.

We examined GHRL expression in relation to sample type (healthy/primary tumor), tumor stage (stages 1–4), and *H. pylori* status by applying UALCAN. As shown in **Figure 2A**, GC samples had lower GHRL expression than healthy samples (*p* = 0.0036). However, there was no significant difference between *H. pylori*-positive and *H. pylori*-negative tumors (**Figure 2B**). A study of tumor stages revealed that GHRL in the middle and late-stage cancers was significantly higher expressed than in the early stages, suggesting a potential function for GHRL in cancer development and migration **(** **Figure 2C**).

**Figure 3:**
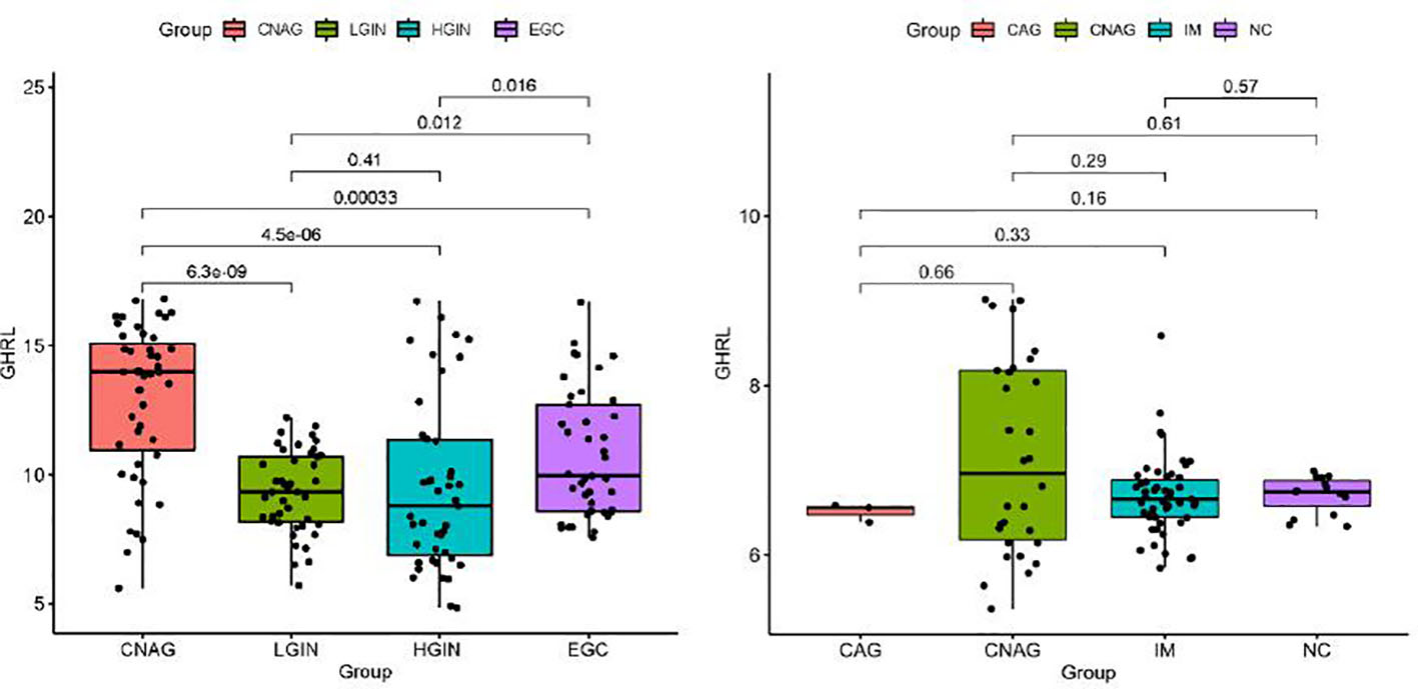
The GHRL expression in different stages of GC Correa’ cascade. CNAG, chronic non-atrophic gastritis; LGIN, low-grade intraepithelial neoplasia; HGIN, high-grade intraepithelial neoplasia; EGC, early GC; CAG, chronic atrophic gastritis; IM, intestinal metaplasia; NC, normal control.

To further analyze the dynamic changes of GHRL in different pathological stages of GC Correa’ cascade, we used R software to analyze the GEO dataset. We found that the expression of GHRL in GC was higher than that in dysplasia (LGIN+HGIN), and the expression of GHRL in dysplasia was significantly lower than that in CNAG (**Figure 3A**). However, there was no significant difference in GHRL expression among CAG, CNAG, and NC (**Figure 3B**).

### GHRL expression is linked to poor overall survival in GC patients

**Figure 4:**
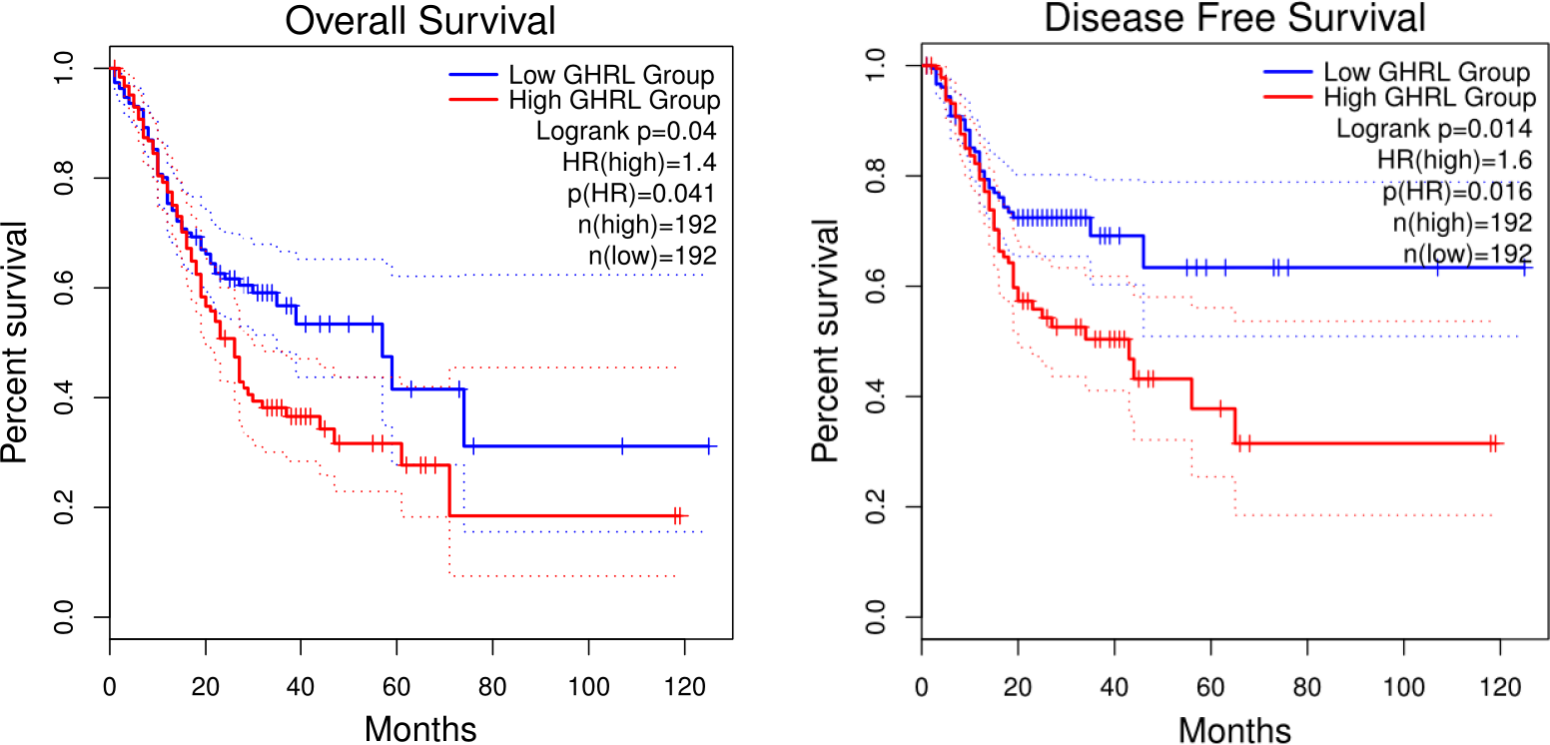
Kaplan–Meier survival curves comparing the high and low expression of GHRL in GC in GEPIA databases.

In this research, we evaluate whether GHRL can be used as a prognostic biomarker in GC via Kaplan–Meier survival curves. This study showed that increased GHRL expression was linked with poorer overall survival (OS) and disease-free survival (DFS) in GC (**Figure 4**). To study the significance and possible molecular mechanisms of GHRL expression in tumor development, we found the correlation between the GHRL expression and clinical–pathological features of GC in the KM plotter. High GHRL expression was linked to poorer OS in female patients and poorer PPS in male patients. Specifically, increased GHRL expression was associated with poorer PPS in stage 3 (PPS HR = 0.61, *p*= 0.046) of GC patients (**Table 1**). We also discovered that OS and PPS at stage T3 (OS, HR = 0.62, *p* = 0.0077) and N1 + 2 + 3 (OS, HR = 0.75, *p* = 0.042) were related to GHRL expression. Surely, we found that PPS of intestinal GC based on Lauren classification was related to high GHRL expression. These findings suggested that the prognostic significance of GHRL in GC patients was determined by their clinical features.

**Table 1:** Correlation of GHRL mRNA expression and clinical prognosis in gastric cancer with different clinicopathological factors by Kaplan–Meier plotter.

Clinicopathological characteristics	Overall survival (n = 881)			Post-progression survival (n = 503)		
	N	Hazard ratio	*p*	N	Hazard ratio	*p*
Sex						
Female	187	1.77 (1.07–2.92)	**0.025**	127	1.53 (0.90–2.59)	0.110
Male	349	1.27 (0.90–1.78)	0.170	256	0.66 (0.45–0.97)	**0.035**
Stage						
1	62	2.21 (0.68–7.21)	0.180	31	5.12 (0.61–42.56)	0.092
2	135	0.65 (0.33–1.30)	0.220	105	1.42 (0.74–2.74)	0.290
3	197	0.78 (0.53–1.14)	0.190	142	0.61 (0.38–1.00)	**0.046**
4	140	0.75 (0.50–1.14)	0.180	104	1.48 (0.85–2.56)	0.160
Stage T						
2	241	1.45 (0.85–2.47)	0.170	196	1.44 (0.91–2.28)	0.120
3	204	0.62 (0.44–0.88)	**0.008**	150	0.68 (0.46–1.01)	0.057
4	38	0.64 (0.28–1.48)	0.290	29	3.19 (0.90–11.36)	0.061
Stage N						
0	74	2.05 (0.76–5.53)	0.150	41	3.37 (0.74–15.42)	0.096
1 + 2+3	422	0.75 (0.57–0.99)	**0.042**	337	0.79 (0.58–1.09)	0.150
1	225	0.8 (0.53–1.22)	0.300	169	1.39 (0.86–2.24)	0.180
2	121	0.65 (0.41–1.03)	0.067	105	0.63 (0.38–1.07)	0.086
3	76	0.65 (0.36–1.15)	0.140	63	1.57 (0.75–3.26)	0.230
Stage M						
0	444	0.79 (0.59–1.05)	0.100	342	0.78 (0.56–1.09)	0.140
1	56	0.69 (0.37–1.31)	0.260	36	0.69 (0.33–1.40)	0.300
Lauren classification						
Intestinal	269	1.57 (1.00–2.49)	0.051	192	1.79 (1.04–3.07)	**0.034**
Diffuse	240	0.74 (0.52–1.04)	0.085	176	0.77 (0.53–1.14)	0.190
Mixed	29	2.36 (0.74–7.52)	0.130	–	–	–
HER2						
Positive	202	1.33 (0.89–2.00)	0.160	101	0.58 (0.32–1.07)	0.079
Negative	429	1.29 (0.94–1.78)	0.120	283	1.29 (0.87–1.92)	0.210

Bold values indicate p < 0.05.

### Correlation between immune infiltration and GHRL expression in GC

**Figure 5:**
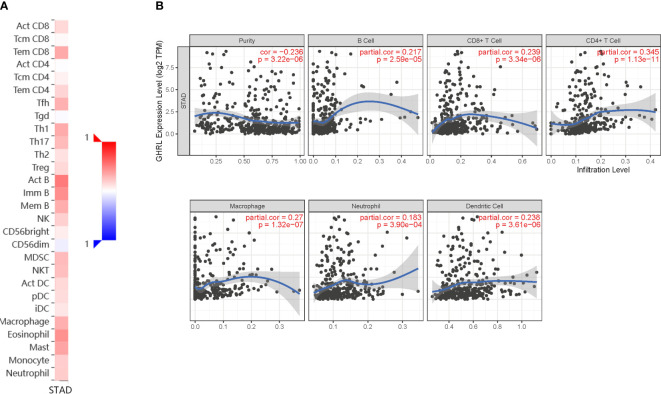
Correlation of GHRL expression with immune infiltration in GC. **(A)** Correlation between the expression of GHRL and the abundance of TILs in GC available at TISIDB database. **(B)** Correlation of GHRL expression with infiltration levels of B cell, CD8 + T cell, CD4 + T cell, macrophage, neutrophil, and dendritic cell in GC available at TIMER2.0 database. TILs, tumor-infiltrating lymphocytes.

Immune infiltration is an important factor associated with tumor development (34). In this study, TISIDB and TIMER platforms were used to assess GHRL expression in connection with immune cell infiltration levels in GC. GHRL expression was adversely correlated with the purity of STAD (rho = −0.236, *p <*3.22e−6). Our results also discovered that GHRL had a correlation with the abundance of TILs (**Figure 5A**). High expression level of GHRL was positively correlated with infiltrating degree of B cells (rho = 0.217), CD8+ T cells (rho = 0.239), CD4+ T cells (rho = 0.345), macrophages (rho = 0.27), neutrophils (rho = 0.183), and dendritic cells (rho = 0.238) **(** **Figure 5B**). These results indicate that GHRL plays an important function in the immune infiltration of GC. TIMER and GEPIA databases were used to study the association between GHRL and different biomarkers of TILs (CD8+ T cells, B cells, T cells, NK cells, monocytes, DCs, TAMs, M1macrophages, M2 macrophages, neutrophils, and related subtypes) in STAD. We found that GHRL was associated with the majority of TILs markers in STAD. GHRL was linked to the majority of immune marker sets of TILs in STAD (**Table 2**). Indeed, GHRL had a significant association with the majority of marker sets of CD8+ T cells, B cells, monocytes, M2 macrophages, DCs, Th2, Th17, Treg, and T-cell exhaustion in STAD (**Table 2**), including CD8A, CD8B, CD19, CD86, CD163, BDCA-1(CD1C), GATA3, STAT6, STAT3, FOXP3, CCR8, CTLA4, and LAG3 (*p*<0.01). Furthermore, according to the GEPIA database, we further assessed the connection of GHRL expression with the markers of M1 macrophages, M2 macrophages, monocytes, TAMs, and T-cell exhaustion (**Table 3**). Therefore, GHRL may regulate T-cell exhaustion and macrophage polarization in GC.

**Table 2:** Correlation analysis between GHRL and related genes and markers of immune cells in Tumor Immune Estimation Resource (TIMER2.0).

Description	Gene markers	STAD	
		Cor	*p*
CD8^+^ T cell	CD8A	0.271	****
	CD8B	0.251	***
B cell	CD19	0.439	***
Monocyte	CD86	0.224	**
TAM	CD68	0.154	0.003
M1 Macrophage	INOS(NOS2)	0.024	0.640
M2 Macrophage	CD163	0.150	*
Natural killer cell	KIR3DL1	-0.028	0.580
Dendritic cell	BDCA-1(CD1C)	0.525	***
Th2	GATA3	0.267	***
	STAT6	0.132	**
Th17	STAT3	0.140	*
Treg	FOXP3	0.233	***
	CCR8	0.230	***
Tcell exhaustion	CTLA4	0.148	*
	LAG3	0.160	*

STAD, stomach adenocarcinoma; TAM, tumor-associated macrophage; Th, T helper cell; Tfh, follicular helper T cell; Treg, regulatory T cell; R, R-value of Spearman’s correlation

*p < 0.01; **p < 0.001; ***p < 0.0001.

**Table 3:** Correlation analysis between GHRL and related genes and markers of immune cells in Gene Expression Profiling Interaction Analysis (GEPIA).

Description	Gene markers	STAD			
		Tumor		Normal	
		R	*p*	R	*p*
Monocyte	CD86	0.170	**	0.640	***
	CD115 (CSF1R)	0.250	***	0.420	0.012
TAM	CCL2	0.120	0.015	-0.051	0.770
	CD68	0.110	0.031	0.370	0.028
	IL10	0.180	**	0.530	**
M1 Macrophage	INOS(NOS2)	0.023	0.640	0.250	0.140
	IRF5	0.190	***	0.071	0.680
	COX2(PTGS2)	0.050	0.320	-0.170	0.310
M2 Macrophage	CD163	0.033	0.510	-0.250	0.140
	VSIG4	0.053	0.280	0.050	0.770
	MS4A4A	0.150	*	0.094	0.590
T cell exhaustion	PD-1(PDCD1)	0.170	**	0.740	***
	PDL1	-0.009	0.850	0.080	0.640
	CTLA4	0.100	0.038	0.680	***
	LAG3	0.120	0.013	0.650	***
	TIM-3(HAVCR2)	0.160	*	0.530	**
	GZMB	−0.016	0.750	0.690	***

STAD, stomach adenocarcinoma; TAM, tumor-associated macrophage; R, R-value of Spearman’s correlation.

*p < 0.01; **p < 0.001; ***p < 0.0001.

### The expression of GHRL is associated with immunomodulators in GC

**Figure 6:**
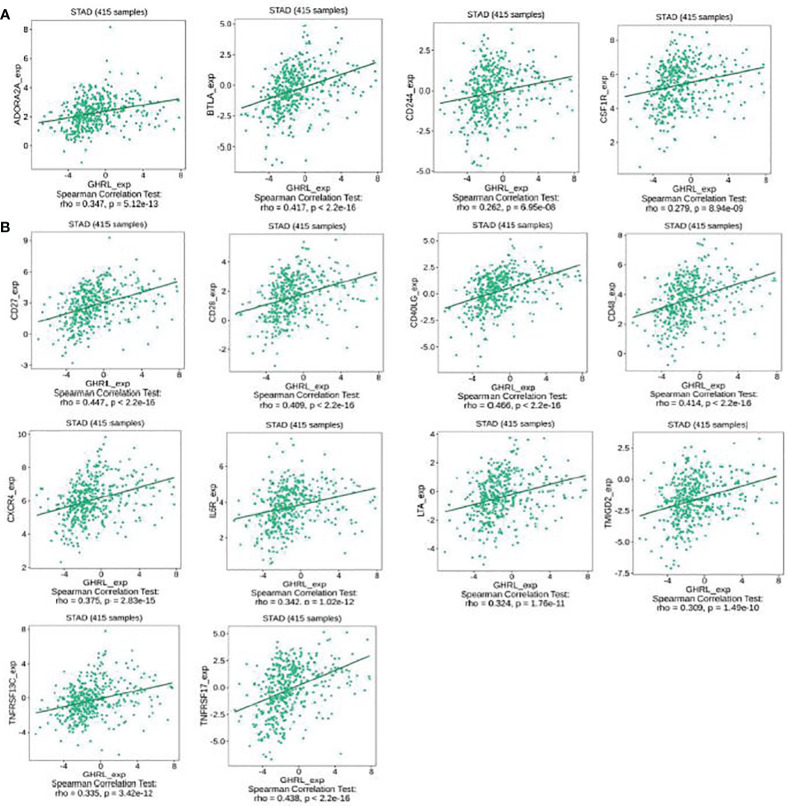
The expression of GHRL is associated with immunomodulators in GC. **(A)** Correlation between GHRL expression and immunoinhibitors in GC available at TISIDB database. **(B)** Correlation between GHRL expression and immunostimulators in GC available at TISIDB database.

This research indicated that GHRL was significantly connected with immunoinhibitors (p < 0.0001), such as ADORA2A (rho = 0.347), BTLA (rho = 0.417), CD244 (rho = 0.262), and CSF1R (rho = 0.279) (**Figure 6A**). The expression of GHRL was also closely associated with immunostimulators (p < 0.0001), including CD27 (rho = 0.447), CD28 (rho = 0.409), CD40LG (rho = 0.466), CD48 (rho = 0.414), CXCR4 (rho = 0.375), IL6R (rho = 0.342), LTA (rho = 0.324), TMIGD2 (rho = 0.309), TNFRSF13C (rho = 0.335), and TNFRSF17 (rho = 0.438) (**Figure 6B**). These results suggested that GHRL is engaged in the regulation of immune interaction and may modulate tumor immune escape.

### Correlation between GHRL expression and chemokines in GC

**Figure 7:**
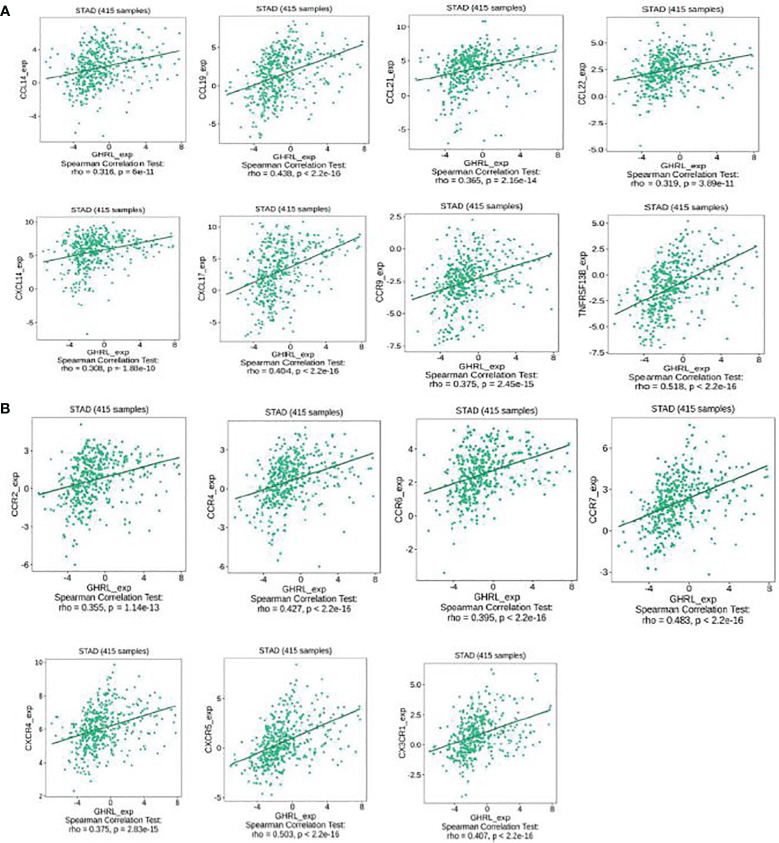
Correlation between the expression of GHRL and chemokines in GC. **(A)** Correlation between GHRL expression and chemokines in GC available at TISIDB database. **(B)** Correlation between GHRL expression and chemokine receptors in GC available at TISIDB database.

This research also implicated the association between GHRL expression with chemokines. For instance, GHRL expression was significantly linked to CCL14 (rho = 0.316), CCL19 (rho = 0.438), CCL21 (rho = 0.365), CCL22 (rho = 0.319), CXCL14 (rho = 0.308), CXCL17 (rho = 0.404), CCR9 (rho = 0.375), and TNFRSF13B (rho = 0.518) (**Figure 7A**). Meanwhile, we demonstrated that GHRL expression was also correlated with chemokine receptors (*p* < 0.001), including CCR2 (rho = 0.355), CCR4 (rho = 0.427), CCR6 (rho = 0.395), CCR7 (rho = 0.483), CXCR4 (rho = 0.375), CXCR5 (rho = 0.503), and CX3CR1 (rho = 0.407) (**Figure 7B**). These results demonstrated that GHRL may function as an immunoregulatory factor in GC.

### Protein–protein interaction network construction and enrichment analysis

**Figure 8:**
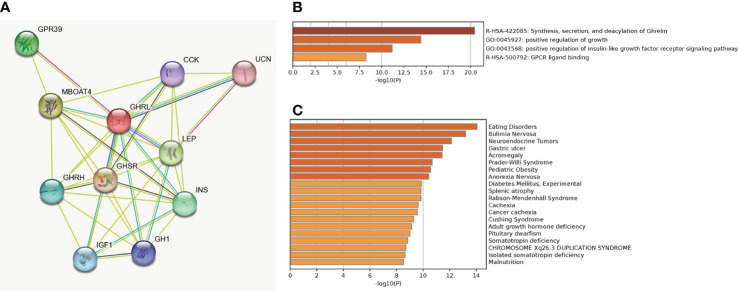
PPI network based on string database **(A)**, biological process enrichment analysis of GHRL-related genes **(B)**, and enrichment analysis on human disease **(C)**.

A total of 11 genes (GHRL, GHRH, GHSR, CCK, GPR39, MBOAT4, UCN, LEP, INS, GH1, and IGF1) were filtered into the target genes PPI network complex, containing 11 nodes and 38 edges (**Figure 8A**). The enrichment analysis indicated that co-expressed genes of GHRL were highly enriched in synthesis, secretion, and deacylation of Ghrelin, positive regulation of growth, positive regulation of insulin-like growth factor receptor signaling pathway, GPCR ligand binding (**Figure 8B**), eating disorders, bulimia nervosa, neuroendocrine tumors, and gastric ulcer (**Figure 8C**).

### GHRL expression in GC tissues

**Figure 9:**
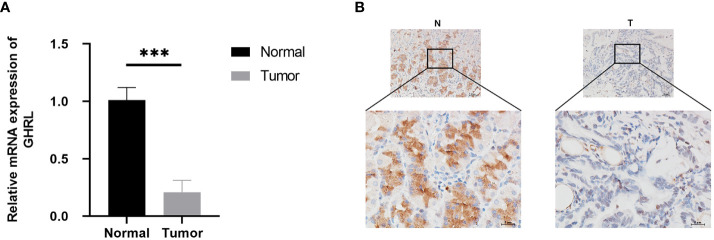
GHRL expression in nine pairs of GC tissues performed by qRT-PCR **(A)** and IHC **(B)**, ***p < 0.0001.

In order to characterize GHRL expression in GC tissues, qRT-PCR was performed, and the results showed that GHRL expression was significantly lower in GC tissues than adjacent noncancerous tissues (**Figure 9A**). In addition, we conducted an IHC method to determine that GHRL expression was lower in GC tissues than those in adjacent non-cancerous tissues **(** **Figure 9B** **)**.

## Discussion

In the present study, a comprehensive bioinformatics investigation was performed to analyze the clinical significance and expression level of GHRL in GC. Our results revealed that poor prognosis was consistent with high expression of GHRL in GC. Furthermore, our data also indicated that GHRL expression had a close association with the infiltration degrees of different immune cells, immunostimulators, immunoinhibitors, chemokines, and receptors in GC. Therefore, our research revealed new insights in understanding the function of GHRL, and it may be a prognostic biomarker linked to immune infiltration of GC.

Ghrelin, a widely distributed peptide hormone, participates in a series of cancer progression (35); GHRL plays a vital role in carcinogenic potential, which was correlated with breast cancer, lung cancer, bladder cancer, and adrenal cancer (17–20). There is an increasing evidence implicating an immunoregulatory role for ghrelin. Ghrelin mainly acts on the innate and adaptive immune systems to suppress inflammation and induce an anti-inflammatory profile (36). However, the possible function of GHRL in regulating tumor immunity and its clinical significance in GC are still unknown.

Therefore, we evaluated GHRL expression in GC using databases including GEPIA, TIMER, GEO, and UALCAN. We discovered that GHRL was clearly decreased in GC compared with normal samples. These results showed that the level of GHRL expression may serve as a potential diagnostic biomarker in GC. Furthermore, to confirm whether GHRL can be used as a prognostic biomarker, we used the KM plotter database to analyze the correlation between GHRL expression and OS and PPS. However, the results indicated that the higher GHRL expression correlated with worse OS and poor PPS of GC. In addition, high GHRL expression had a significant correlation with a worse prognosis of GC in stages T3 and N1 + 2 + 3 for worse OS and in stage 3 for worse PPS. These observations support our hypothesis that GHRL may act as an anti-oncogene in GC. For low expression of GHRL in GC and high expression of GHRL linked with poor prognosis in GC, previous studies have also found similar seemingly contradictory results, and they made no explanation and experimental verification (37–39). Our explanation may be that the higher is the malignancy, the more oncogene GHRL is needed in GC. In addition, previous studies found that the expression of GHRL was decreased in intestinal GC compared with diffuse histotype via transcriptome analysis on GC specimens (40). In addition, we first discovered that the expression of GHRL in dysplasia was significantly lower than that in CNAG and in GC, so we speculated that ghrelin may be associated with the progression of precancerous lesions. GHRL played different roles in different pathological stages. Previously, a large number of scholars studied the relationship among ghrelin, gastric mucosal atrophy, and *H. pylori* status, but the results were controversial (41–46). Our study first discovered that GHRL was not related to gastric mucosal atrophy and *H. pylori* status via bioinformatics investigation, probably because of the small sample size of GEO database on gastric mucosal atrophy; therefore, large sample clinical studies and fundamental experiment may be needed for validation in the future.

Additionally, this study discovered that GHRL was related to immune infiltration in GC. In the tumor microenvironment, it has been demonstrated that immune cell infiltration played vital roles in the development and progression of cancer (47, 48). GHRL played a role in inflammatory and immune responses (49). Recent studies have revealed that GHRL was involved in the regulation of metabolism, energy balance, and the immune, cardiovascular, and reproductive systems (50–52). However, whether GHRL expression was linked to immune infiltration in GC remains unknown. Therefore, we systematically examined the association between GHRL expression and the degree of immune infiltration in GC. In our study, this was the first time that GHRL regulating immune infiltration in GC was identified. Our study showed that GHRL expression had a correlation with TILs including B cells, CD8+ T cells, CD4+ T cells, macrophages, neutrophils, and dendritic cells. At the same time, decreased GHRL expression was associated with immunostimulators, immunoinhibitors, chemokines, and receptors. In addition, this study also demonstrated the association between GHRL expression and the TIL marker genes of GC. Indeed, GHRL expression had an association with M1 macrophage markers, like IRF5, and M2 macrophage markers, such as MS4A4A and CD163, correlated with GHRL expression. These findings indicate that GHRL has a potential function to regulate macrophage polarization.

Furthermore, our results revealed that GHRL expression was significantly correlated to cell response to chemokines according to the TISIDB databases. These results reflected that it may be a direction for enhancing immunotherapy effectiveness by targeting GHRL. Above all, GHRL played a key function in recruiting and modulating TILs in GC, and it is worth to continue investigating the molecular mechanism and function of GHRL in modulating the tumor microenvironment.

Unfortunately, we are yet to conduct experimental studies validating the function of GHRL in the development of GC and the molecular mechanism of GHRL in GC immunity, but in the future, we guarantee that experiments will be performed to further validate the projected results.

## Conclusion

The high GHRL expression is closely correlated with poor prognosis. GHRL may be associated with the progression of precancerous lesions in GC. The low GHRL expression enhanced immune infiltration degree including B cells, CD8+ T cells, CD4+ T cells, macrophages, neutrophils, and dendritic cells in GC. Moreover, the expression of GHRL contributes to the regulation of CD8+ T cells, B cells, monocytes, M2 macrophages, DCs, Th2, Th17, Treg, and T-cell exhaustion. Therefore, this study suggests that GHRL may serve as a useful biomarker and therapeutic target for patients with GC.

## Preprint

A preprint has previously been published on https://doi.org/10.21203/rs.3.rs-1790354/v1.

## Data availability statement

The original contributions presented in the study are included in the article/supplementary material. Further inquiries can be directed to the corresponding author.

## Ethics statement

The studies involving human participants were reviewed and approved by The Ethics Committee approved the study of the First Affiliated Hospital of Nanchang University (Ethical Application Ref: 2021006). The patients/participants provided their written informed consent to participate in this study.

## Author contributions

YX designed the experiment and supervised the study. DL and JW contributed to formal analysis. JW wrote the manuscript. YX reviewed and revised the manuscript. All authors contributed to the article and approved the submitted version.

## Footnotes

1 The Tumor Immune Estimation Resource (TIMER2.0) (https://timer.cistrome.org/).

2 The Gene Expression Profiling Interactive Analysis2.0 (GEPIA2) database (http://gepia.cancer-pku.cn/index.html).

3 UALCAN website (https://ualcan.path.uab.edu/).

4 Gene Expression Omnibus (GEO, https://www.ncbi.nlm.nih.gov/geo/).

5 the KM plotter (http://kmplot.com/analysis/).

6 TISIDB (http://cis.hku.hk/TISIDB/index.php).

7 STRING (http://string-db.org).

8 Metascape (https://metascape.org/).
